# Supplementary material for: Ambient noise differential adjoint tomography reveals fluid-bearing rocks near active faults in Los Angeles
Source: Nat Commun. 2023 Oct 28;14:6873. doi: 10.1038/s41467-023-42536-4 (PMC10613219; doi:10.1038/s41467-023-42536-4)
Supplement: Supplementary file 1 — Supplementary Information [file 41467_2023_42536_MOESM1_ESM.pdf]

Supporting Information for

Fluid-bearing rocks near active faults in the urban Los Angeles basin revealed by ambient noise differential adjoint tomography

Xin Liu[1,2,3*], Gregory C. Beroza[2*] and Hongyi Li[3,4]

Correspondence to:
Xin Liu: liuxine@hku.hk
Gregory C. Beroza: beroza@stanford.edu

Contents of this file

Figure S1
Figure S2
Figure S3
Text S1
Text S2
Table S1

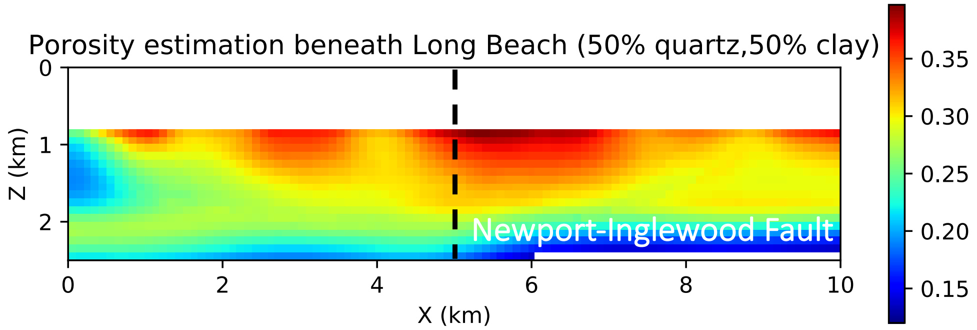

Figure S1. The lower bound of porosity converted from Vp/Vs ratio using BGTL method. At 0.8 km depth, the porosity is exact. For depth below 0.8 km, the displayed porosity value is a lower bound.

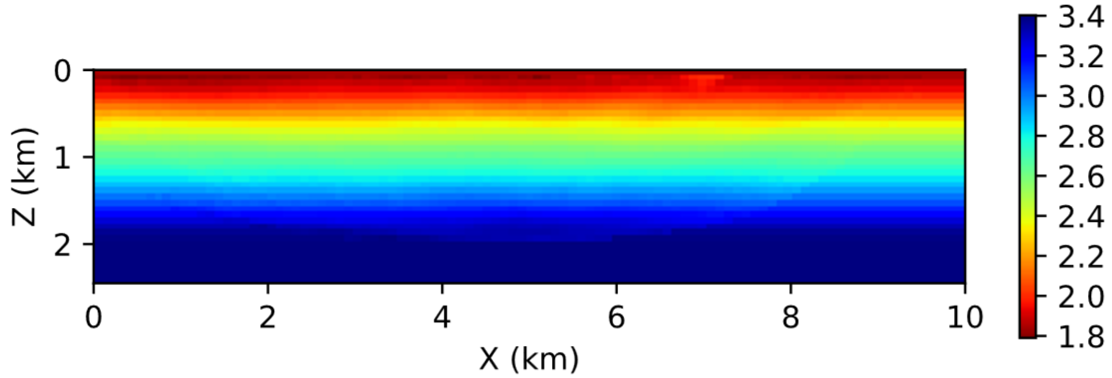

Figure S2. P wave velocity model (Castellanos et al., 2020).

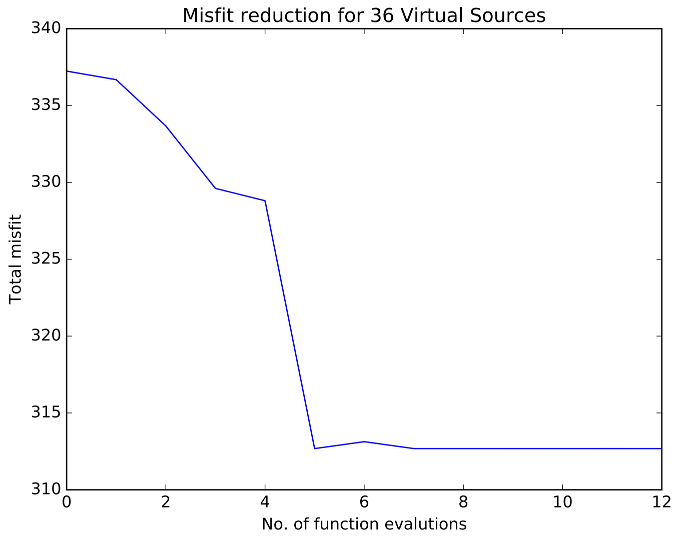

Figure S3. Misfit function versus iterations for low+high frequency signals.

Text 1. Adjoint sources for differential time measurements on dispersive signals

We follow Yuan et al. (2016) and Liu (2020) for the definition of differential time misfit functions. For one station triplet (Fig. 5B), the differential time misfit function is,

$$\chi_{ij}^{dd} = \frac{1}{2}\sum_{\omega} W(\omega)\left[\Delta t_{ij}(\omega) - \Delta t_{ij}^{obs}(\omega)\right]^2, \qquad \text{(S1)}$$

where $\Delta t_{ij}^{obs}(\omega)$ and $\Delta t_{ij}(\omega)$ are, respectively, observed and synthetic differential time measurements between station pairs 1-2 and 1-3 ($i \equiv$ station 2 ; $j \equiv$ station 3). Here $W(\omega)$ is a weighting function and ω is angular frequency.

In the frequency domain, we define the data and synthetic waveform as $d_i(\omega)$ and $u_i(\omega)$, respectively. The synthetic waveform is formulated in terms of amplitude $A_i(\omega)$ and phase $t_i(\omega)$ terms,

$$u_i(\omega) = A_i(\omega)\exp\left[-i\omega t_i(\omega)\right], \tag{S2}$$

By taking the spectral ratio of two synthetic waveforms, we define the transfer function,

$$\mathrm{T}_{ij}(\omega) = \frac{u_i(\omega)}{u_j(\omega)} = \exp\left[\ln A_{ij}(\omega) - i\omega\Delta t_{ij}(\omega)\right], \tag{S3}$$

where $A_{ij}(\omega)=A_i(\omega)/A_j(\omega)$, and $\Delta t_{ij}(\omega)=t_i(\omega)-t_j(\omega)$.

Taking the variation of eq. (S3), we have,

$$\delta\mathrm{T}_{ij}(\omega) = \frac{\delta u_i(\omega)}{u_j(\omega)} - \frac{u_i\delta u_j(\omega)}{u_j^2(\omega)} = \mathrm{T}_{ij}(\omega)\left[\delta\ln A_{ij}(\omega) - i\omega\delta\Delta t_{ij}(\omega)\right]. \tag{S4}$$

We subtract the complex conjugate of eq. (S4) from eq. (S4) to cancel the amplitude $A_{ij}(\omega)$ terms. Rearranging the terms and dividing by $2i\omega\mathrm{T}_{ij}(\omega)$, we obtain the expression for perturbation in differential time,

$$\delta\Delta t_{ij}(\omega) = \frac{i}{2\omega}\left[\frac{\delta u_i(\omega)}{u_i(\omega)} - \left(\frac{\delta u_i(\omega)}{u_i(\omega)}\right)^* - \frac{\delta u_j(\omega)}{u_j(\omega)} + \left(\frac{\delta u_j(\omega)}{u_j(\omega)}\right)^*\right]. \tag{S5}$$

where the superscript * represents complex conjugate.

The variation of misfit function for a station triplet is,

$$\delta\chi_{ij}^{dd} = \sum_{\omega} W(\omega)\left[\Delta t_{ij}(\omega) - \Delta t_{ij}^{obs}(\omega)\right]\delta\Delta t_{ij}(\omega). \tag{S6}$$

To convert the variation of misfit function to the time domain, we apply Parseval's theorem and Inverse Fourier Transform,

$$\begin{aligned}\delta\chi_{ij}^{dd}\left(T_{\max}\right) = 4\pi\int_0^{T_{max}} & F^{-1}\left\{W\left(\omega\right)\left[\Delta t_{ij}\left(\omega\right) - \Delta t_{ij}^{obs}\left(\omega\right)\right]p_{i|j}\left(\omega\right)\right\}\delta u_i\left(t\right)dt \\ & + F^{-1}\left\{W\left(\omega\right)\left[\Delta t_{ij}\left(\omega\right) - \Delta t_{ij}^{obs}\left(\omega\right)\right]p_{j|i}\left(\omega\right)\right\}\delta u_j\left(t\right)dt\end{aligned} . \tag{S7}$$

where $p_{i|j}$ and $p_{j|i}$ are defined as,

$$\begin{aligned} p_{i|j}\left(\omega\right) &= \frac{i}{2\omega}\frac{1}{u_i\left(\omega\right)} = \frac{i}{2\omega}\frac{1}{A_i\left(\omega\right)}\frac{u_i^*\left(\omega\right)}{\left|u_i\left(\omega\right)\right|} \\ p_{j|i}\left(\omega\right) &= \frac{-i}{2\omega}\frac{1}{u_j\left(\omega\right)} = \frac{-i}{2\omega}\frac{1}{A_j\left(\omega\right)}\frac{u_j^*\left(\omega\right)}{\left|u_j\left(\omega\right)\right|} \end{aligned} . \tag{S8}$$

Eq. (S8) contains the complex conjugate phase factor of the synthetic waveform. Therefore, we do not apply multi-taper analysis for the synthetic waveform. An appropriate bandpass filter is used to remove the large amplitude in the transition band.

Based on eqs. (S7 & S8), we define the adjoint sources for dispersive signals of one station triplet as,

$$\begin{aligned} f_i^\dagger\left(T-t\right) &= 4\pi F^{-1}\left\{W\left(\omega\right)\left[\Delta t_{ij}\left(\omega\right) - \Delta t_{ij}^{obs}\left(\omega\right)\right]p_{i|j}\left(\omega\right)\right\}\left(t\right)\delta\left(\mathbf{x}-\mathbf{x}_i\right) \\ f_j^\dagger\left(T-t\right) &= 4\pi F^{-1}\left\{W\left(\omega\right)\left[\Delta t_{ij}\left(\omega\right) - \Delta t_{ij}^{obs}\left(\omega\right)\right]p_{j|i}\left(\omega\right)\right\}\left(t\right)\delta\left(\mathbf{x}-\mathbf{x}_j\right) \end{aligned} . \tag{S9}$$

where the term T-t means time reversal of the right-hand side.

For a virtual source denoted as 1, it is convenient to define a misfit function summing over all eligible station triplets,

$$\chi^{dd} = \sum_{i,j\in\Omega}\chi_{ij}^{dd} , \tag{S10}$$

where Ω contains the i,j pairs such that the distance 1-j is slightly longer than 1-i and they are in the (almost) same direction. Because of the linearity in eq. (S10), the adjoint sources of each station triplet are linearly added to corresponding stations.

Text 2. Deriving porosity from BGTL method

We find that the lithology type in the top 2 km depth is primarily sand/silt/clay (Ponti et al., 2007) near the LBCC borehole in the Long Beach area. For simplicity, we assume that the matrix material is half quartz and half clay in volume. In Table S1, we list the parameters used in eq. (8) of Lee (2003), where we use n=1.

Parameter	Value
Shear modulus of quartz	45 Gpa
Bulk modulus of quartz	36 Gpa
Shear modulus of clay	6.85 Gpa
Bulk modulus of clay	20.9 Gpa
Density of quartz	2.65 g/cm^3

Table S1. Parameters used for converting Vp/Vs ratio to porosity using BGTL method.
